# A Model-to-Monitor Evaluation of 2011 National-Scale Air Toxics Assessment (NATA)

**DOI:** 10.3390/toxics7010013

**Published:** 2019-03-10

**Authors:** Zhuqing Xue, Chunrong Jia

**Affiliations:** School of Public Health, University of Memphis, Memphis, TN 38152, USA; zxue@memphis.edu

**Keywords:** hazardous air pollutant, model-to-monitor comparison, NATA, modeling, model performance

## Abstract

Environmental research has widely utilized the ambient concentrations of hazardous air pollutants (HAPs) modeled by the National-Scale Air Toxics Assessment (NATA) program; however, limited studies have evaluated the model’s performance. This study aims to evaluate the model-to-monitor agreement of the 2011 NATA data with the monitoring data reported to the U.S. Environmental Protection Agency’s (EPA) Air Quality System (AQS). Concentrations of 27 representative HAPs measured at 274 sites in the U.S. in 2011 were merged with NATA data by census tract. The comparison consisted of two steps for each HAP: first, the model-monitor difference at each site was compared with the limit of quantitation (LOQ); second, the modeled annual average was compared to the 95% confidence interval of the monitored annual average. Nationally, NATA could predict national medians of most HAPs well; however, it was unable to capture high concentrations. At individual sites, a large portion of model-monitor differences was below the LOQs, indicating they were unquantifiable. Model-to-monitor agreement displayed inconsistent patterns in terms of chemical groups or EPA regions and was strongly impacted by the comparison methods. The substantial non-agreements of NATA predictions with monitoring data require caution in environmental epidemiology and justice studies that are based on NATA data.

## 1. Introduction

Valid and representative data of hazardous air pollutants (HAPs) are required to evaluate emission compliance, air quality attainment, and population health risks. Chronic and acute exposure to HAPs may cause damage to multiple human organs [1], including respiratory [2], nervous [3,4], circulatory [5], reproductive [6], immune [7], digestive [8], and urinary systems [9]. The U.S. Environmental Protection Agency (EPA) aimed to reduce HAP emissions by 75% of the 1993 level to meet the requirements of the Government Performance and Results Act. EPA has been working with state, local and tribal air pollution control agencies to measure ambient HAP concentrations. The current monitoring efforts are inadequate for increasingly refined health and climate studies. Health data are collected at the individual level or small geographic scale; however, sparsely distributed air monitoring stations often lack spatial representativeness [10]. For example, national analyses of air pollutants only identified 169 different stations for polycyclic aromatic hydrocarbons (PAHs) [11] and 379 stations for fine particulate matter (PM_2.5_) [12]. The sub-kilometer scale variation of air pollutants requires dense sampling networks with more than 1–2 nodes per km^2^ [13], which far exceed the current capacity. Modeling programs are then developed to estimate exposures at high temporal and spatial resolutions [14].

EPA initiated the National-scale Air Toxics Assessment (NATA) in 1996 to serve as a geographical extension of the existing air monitoring network. NATA is designed to inform decision-making, e.g., to prioritize pollutants and sources, identify locations for investigation, and design monitoring programs [15]. NATA models HAP concentrations at geographic resolutions down to the census tract level. These high spatial-resolution data have many environmental applications. Environmental epidemiology studies have used NATA data to explore associations between HAP exposure and health endpoints such as respiratory disease [16,17], autism spectrum disorder in children [18], and school performance [19,20]. The cancer risk estimates in NATA often serve as bases for addressing environmental justice issues [21,22,23,24,25,26,27,28]. NATA methodology and data are also used to model population exposure [29], predict future exposures [30], estimate excess risks [31], and establish emission-to-intake relationship [32].

Evaluating NATA model performance is imperative given the numerous applications of NATA data. NATA modeling uses conservative assumptions that potentially lead to overestimation [15]; however, some comparison studies gave the opposite results [33,34,35]. A few independent evaluation studies used local-scale monitoring in California [35], Pittsburgh, Pennsylvania [33], Detroit, Michigan [36], Texas [34], and South Baltimore, Maryland [37]. These model-to-monitor comparisons are often limited in terms of the number of chemicals and geographic areas. EPA has conducted limited evaluations and encouraged more studies [36,38].

The 2011 NATA yielded a rich database that contains concentrations, exposures, and cancer and non-cancer risks for 180 HAPs, as well as their contributing sources. There has been no independent evaluation of 2011 NATA, although EPA has made limited model-to-monitor comparisons for selected compounds [39]. Methodologically, EPA used multiple comparison measures, e.g., linear regressions, a factor of 2, and absolute biases [40]; however, they often give inconsistent results. Measurement uncertainty was not considered in previous comparisons, which might lead to massive biases as many modeled concentrations are far below the detection limits. These limitations call for a systematic approach for model-to-monitor comparisons.

This study aims to evaluate the model performance of 2011 NATA by comparing modeling and monitoring concentrations. We compile the real measurements collected at 274 sites throughout the U.S. in 2011 and merge modeling and monitoring datasets. We then assess agreement using statistical and empirical methods, considering the measurement uncertainty.

## 2. Materials and Methods

### 2.1. Data Sources and Compilation

The monitoring HAP data were extracted from the U.S. EPA’s Air Quality System (AQS). AQS is a web-based air pollution database accessible to the public. It contains ambient air pollution data and sampling condition information collected from tribal, local and state agencies through consistent and strict quality assurance (QA) processes. The HAPs were monitored following EPA’s Air Toxics Monitoring Methods [41]. In brief, volatile organic compounds (VOCs) were measured by the TO-15 method, aldehydes by TO-11A, PAHs by TO-13A and heavy metals (including antimony, arsenic, beryllium, cadmium, chromium, cobalt, lead, manganese, nickel and selenium) by the IO-3.5 method. Most HAP samples were analyzed at central laboratories and their typical limits of quantitation (LOQs) are available [42,43]. We downloaded daily (24-hour) HAP concentrations measured in 2011 [44]. Conventional units, such as part per billion (ppb), part per million (ppm), ppb carbon (ppbC), or ppm carbon (ppmC), were converted to the standard unit µg/m^3^ to match that used in NATA. Locations of the monitoring sites in AQS were geocoded and assigned the census tract number in ArcGIS (v10.3.1, ESRI Inc., Redlands, CA, USA).

**Figure 1:**
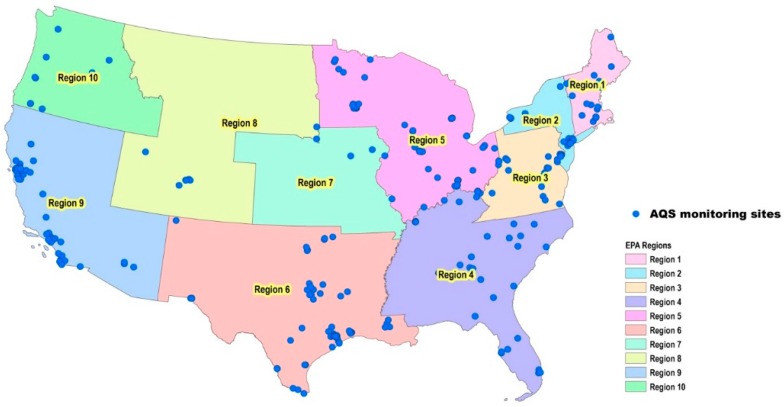
Air toxics monitoring sites archived in the U.S. Environmental Protection Agency (EPA)’s Air Quality System (AQS).

The modeling HAP concentrations at the census tract level were downloaded from the 2011 NATA database. The 2011 NATA contained 78,000 census tracts in the continental U.S. AQS and NATA data were then merged by census tract. The merged dataset contained up to 274 monitoring stations from AQS (Figure 1) but only 274 matched census tracts from NATA. This subset of NATA data was representative of the entire 2011 NATA dataset, as their key descriptive statistics were very similar (Table 1). Thus, NATA in the following text means the matched sub-dataset. In any census tract, NATA gives a single annual average concentration of a HAP, and AQS gives 5–162 measurements of the same HAP taken in the year 2011.

### 2.2. Hazardous Air Pollutants (HAPs) of Interest

We selected 27 HAPs to evaluate the model-to-monitor agreement. The selection was based on four criteria: (1) they had high rankings in both cancer and respiratory risks in NATA; (2) they were measured and detected at ≥25 monitoring sites in AQS; (3) they were prioritized in previous NATA reports; and (4) they represented different chemical groups. We then divided the 27 HAPs into four groups mainly based on their chemical structures: (1) Aromatic compounds: benzene, isopropyl benzene, ethyl benzene, styrene, toluene, 1,4-dichlorobenzene and naphthalene; (2) halogenated compounds: 1,1,2-trichloroethane, bromoethane, carbon tetrachloride, chloroform, methyl chloride, ethylene dichloride, trichloroethylene, tetrachloroethylene and vinyl chloride; (3) carbonyl compounds: methyl isobutyl ketone, acetaldehyde, and formaldehyde; and (4) other compounds: chromium, lead, carbon disulfide, 2,2,4-trimethylpentane, *n*-hexane, 1,3-butadiene, methyl *tert*-butyl ether and acrylonitrile. Not all the sites measured all the compounds and thus the site numbers varied from 27 to 274 depending on the compound.

### 2.3. Model-to-Monitor Comparison Methods

The 2011 NATA modeling results contain very low concentrations for certain compounds, e.g., the annual average concentrations of 1,1,2-trichloroethane and chromium were 0.00041 and 0.00003 µg/m^3^, respectively. In practice, the measurement method has a limit of quantification (LOQ) for a specific chemical. The LOQ is defined as the lowest concentration that can be accurately measured during regular laboratory analyzing conditions [42]. Following the concept of LOQ, the absolute value of difference (ΔM) between modeled concentration (C_NATA_) and monitored concentration (C_AQS_), i.e., ΔM = |C_NATA_ − C_AQS_|, is unquantifiable if ΔM is less than LOQ. C_NATA_ and C_AQS_ can be considered to be in agreement given an unquantifiable ΔM [45].

To evaluate the national-level agreement, we compared national medians rather than means, considering the substantial spatial heterogeneity among monitoring sites. For each HAP, we first determined whether the ΔM was quantifiable, and then compared two medians using the Wilcoxon signed-rank test. A *p*-value of ≥0.05 was considered the agreement.

At individual sites, we compared annual modeled and monitored averages using statistical methods if ΔM was quantifiable. We calculated the 95% confidence interval (CI) of the annual mean concentration of a compound, and then determine if the single NATA annual average value fell within this 95% CI. We log-transformed AQS data as they followed a skewed lognormal distribution, and then calculated the 95% CI using the Cox’s method [46]. This is a strict statistical comparison method and applies the widely accepted criterion of 95% CI or equivalently the *p*-value of 0.05. EPA indicated that statistical analysis was the best way for model performance evaluations [40,47].

At each site, if NATA agreed with the AQS, the site was defined as an agreement site for that chemical; otherwise, it was defined as underestimation or overestimation site. These steps were repeated for all the available sites and the 27 HAPs of interest. The percentages of underestimation, agreement, and overestimation sites were calculated for all the sites in the U.S. as well as by EPA region. While there is not a bright line to define the degree of agreement, we define a compound as an under-predicted, agreement, or over-predicted compound if it is under-predicted, in agreement, or over-predicted at ≥50% of sites, respectively. This definition enables us to get an overall impression of the model-to-monitor agreement of each HAP at the national or regional level.

EPA has long been using a factor of 2 as the criterion for model-to-monitor comparisons [35,48,49], i.e., a C_NATA_/C_AQS_ ratio of 0.5–2 could be considered the agreement. Rather than using the simple ratio, we adopted an equivalent metric fractional bias (FB):

(1) $$\mathrm{FB}=\left[\frac{C_{\mathrm{NATA}}-C_{\mathrm{AQS}}}{\left(C_{\mathrm{NATA}}+C_{\mathrm{AQS}}\right)/2}\right]$$

FB is a relative bias that combines bias and ratio. It is symmetrical, bounded and dimensionless [47]. An FB between −0.67 and +0.67 indicates acceptable agreement, and values of −2 and +2 indicate extreme underestimation and overestimation, respectively [47,50]. An FB was calculated when ΔM = |C_NATA_ − C_AQS_| was quantifiable.

All the analyses were performed in SAS (v9.4, SAS Institute Inc., Cary, NC, USA), Microsoft Excel 2010 (Redmond, WA, USA) and Arc GIS (v10.3.1, ESRI Inc., Redlands, CA, USA).

## 3. Results

### 3.1. Comparison of National Statistics

The AQS monitoring data showed that ambient HAP concentrations were low in the U.S. (Table 2). The median concentrations ranged from near 0 µg/m^3^ (1,1,2-trichloroethane) to 1.44 µg/m^3^ (acetaldehyde). Eighteen compounds had median concentrations below 0.1 µg/m^3^, 5 compounds between 0.1 and 1 µg/m^3^, and 4 above 1 µg/m^3^. Only three compounds had maxima exceeding 10 µg/m^3^: ethylbenzene (12.6 µg/m^3^), trichloroethylene (16.9 µg/m^3^), and carbon sulfide (29.4 µg/m^3^).

The differences between AQS and NATA medians were small, although they were statistically significant for most compounds. Sixteen HAPs had their ΔMs less than the corresponding LOQs, indicating the differences were too small to be measurable. Naphthalene was the only compound that showed good agreement between AQS and NATA medians when ΔM > LOQ. Out of the remaining 10 compounds, 9 had their national medians underestimated by NATA, and acetaldehyde had its national median overestimated by NATA. Overall, NATA predicted national medians correctly for 17 compounds, underestimated medians for 10 compounds, and overestimated medians for 1 compound.

NATA was unable to capture extreme concentrations. The maximum concentrations in AQS were much higher than those modeled by NATA for all HAPs except for toluene, bromomethane, and methyl isobutyl ketone. This could be explained by the inability of dispersion models to simulate extreme concentrations [38,51].

### 3.2. National Model-to-Monitor Agreement

At individual monitoring sites, the ΔM between NATA and AQS annual averages was first examined for each compound (Table 3). The ΔM of chromium was noticeably below the LOQ at all the sites. Similarly, ΔM was below the LOQ at over 90% of sites for cumene, vinyl chloride, 1,1,2-trichloroethane and methyl *tert*-butyl ether, and at 50–90% of sites for another seven compounds. A total of 12 compounds showed agreement at ≥50% of sites by comparing ΔM to LOQ.

When ΔM was quantifiable, toluene, formaldehyde, acetaldehyde, naphthalene showed agreement at 20–27% of sites, methyl chloride, 1,3-butadiene, tetrachloroethylene, and carbon disulfide showed agreement at 10–20% of sites, and the remaining 20 chemicals all showed agreement at <10% of sites. Therefore, NATA agreed with AQS at a small portion (<30%) of sites nationally at the quantifiable concentration ranges (Table 3).

Taken together, 14 compounds had NATA-AQS agreement at >50% of sites, as highlighted in Table 3. Methyl chloride, chloroform, carbon tetrachloride, carbon disulfide and lead were nationally under-predicted, and toluene, formaldehyde and acetaldehyde were nationally over-predicted. Benzene, ethylbenzene, naphthalene, 1,3-butadiene and *n*-hexane did not show strong patterns.

Better agreement results were observed if adopting EPA’s factor of 2 criterion (Table 4). A total of 21 compounds showed agreement at ≥50% of sites. A significant increase in the numbers of agreement sites occurred for benzene, methyl chloride, carbon tetrachloride, formaldehyde and acetaldehyde. Styrene, tetrachloroethylene and 1,3-butadiene showed agreement at 44–48% of sites. Chloroform, carbon disulfide and lead were underestimated at 72–75% of sites. NATA overestimated concentrations at a small portion of sites for all the compounds. These indicated that the “factor of 2” criterion was more lenient than the statistical comparisons.

### 3.3. Regional Model-to-Monitor Agreement

**Figure 2:**
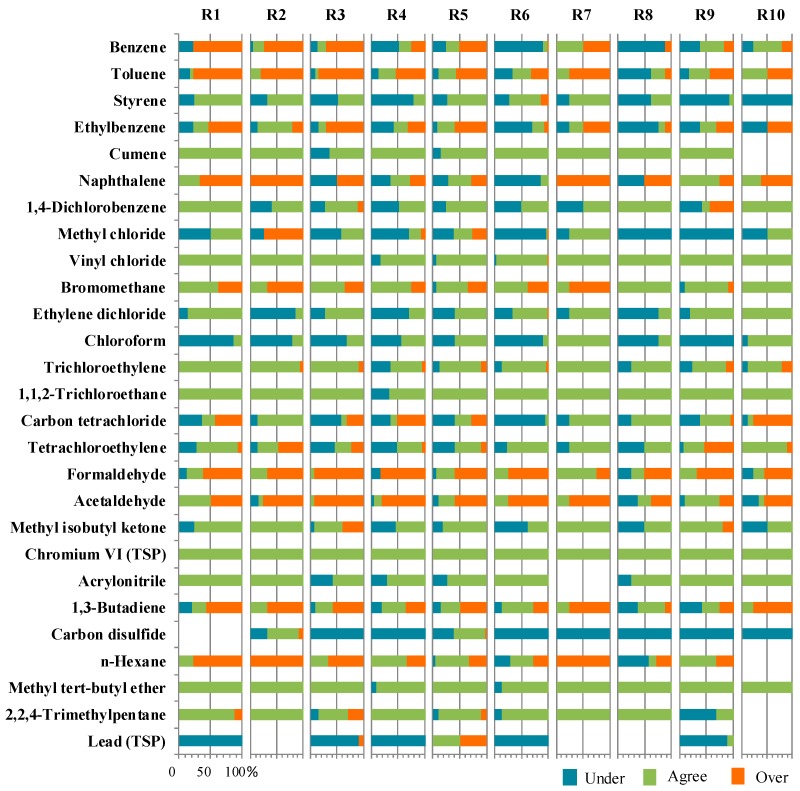
Percentages of agreement, underestimation, and overestimation sites by EPA region. Notes: R—EPA region; Under—underestimation; Agree—agreement; Over—overestimation.

The agreement between NATA estimates and AQS measurements could be further examined by EPA regions, as shown in Figure 2. Checking by compound in Figure 2, lead (TSP), formaldehyde, naphthalene, ethylbenzene, toluene, carbon disulfide, 1,3-butadiene, acetaldehyde, benzene, *n*-hexane, chloroform, methyl chloride and carbon tetrachloride had a poor agreement in most regions. In contrast, eight compounds showed good agreement in all regions, including acrylonitrile, methyl isobutyl ketone, cumene, methyl *tert*-butyl ether, chromium VI (TSP), 1,1,2-trichloroethane, trichloroethylene, vinyl chloride. Checking by region in Figure 1, certain compounds or chemical groups displayed regional characteristics. Aromatic compounds showed poor agreement in Region 1, e.g., benzene did not show model-to-monitor agreement at any sites in Region 1. Halogenated compounds showed poor agreement in Region 2, e.g., methyl chloride did not show model-to-monitor agreement at any sites in Region 2. Carbonyls compounds showed poor agreement in Region 6, as none of the three carbonyls showed agreement at more than 50% of sites. Overall, the agreement displayed a strong by-compound pattern but not a regional pattern.

## 4. Discussion

### 4.1. Similar Findings from National and Local Studies

Our results confirmed previous national and local evaluations of NATA modeling. Previous NATA evaluations found good agreement for only a few compounds and underestimation for most compounds [15,35]. In the 2005 NATA model assessment, only 8 out of 68 compounds showed agreement at the national level, and other compounds were all underestimated [48]. At state and local levels, Lupo and Symanski [34] found 1996 NATA underestimated 8 out 15 HAPs and 1999 NATA underestimated 18 out of 27 HAPs in Texas. Wang et al. [50] found general agreement for benzene and toluene concentrations modeled by 1999 NATA in Camden, New Jersey. Logue et al. [33] reported that the 2002 NATA underestimated 32 out of 49 HAPs measured at 7 sites in and around Pittsburgh, Pennsylvania. The Detroit Exposure and Aerosol Research Study (DEARS) reported that benzene concentrations in 2002 NATA generally agreed with field measurements from 2004 to 2007 [36]. Garcia et al. [35] found that 12 HAPs were underestimated by 1996 NATA, 8 out of 9 were underestimated by 1999 NATA, 10 out of 12 were underestimated by 2002 NATA, and 6 out of 10 were underestimated by 2005 NATA. It was notable that previous studies found good agreement for benzene; however, our results showed moderate agreement, possibly due to different comparison methods. These findings indicate that model-to-monitor agreement was inconsistent by region and chemical, and under-prediction was more frequent [35,36,50]. 

The general underestimation by NATA was attributable to factors including (1) missing emissions sources; (2) underestimated emission rates; (3) sites intended to find peak concentrations; and (4) measurement accuracy [52]. As seen in Table 2, NATA model in general was unable to capture extreme concentrations. Average concentrations measured from monitors within a census tract might be affected by extrema due to nearby short-term strong emissions, which could not be captured by the census-tract averages in NATA. Similarly, the National Emission Inventory, on which NATA estimates were based, might have missed local emission sources [51]. Lack of stable estimates of meteorological conditions and photochemical reactions is another factor leading to disagreement. For example, unstable estimates on wind conditions and secondary formation of chemicals were major weakness of the NATA model [38]. The uncertainty in monitored measurement due to insufficient and unbalanced geographic coverage of monitoring sites also contributed to the discrepancies between monitored and modeled estimates [35,51]. These factors warrant future improvements in both monitoring adequacy (technology, frequency and coverage) and modeling parameterization.

### 4.2. Impacts of Comparison Methods and Metrics

Model-to-monitor comparison results were significantly impacted by comparison methods. We introduced LOQs to overcome the measurement uncertainty issue, which was ignored in previous studies. It turned out model-to-monitor differences were unquantifiable at a large portion of sites for many compounds. A small and practically unquantifiable difference should mean an agreement; however, statistical analyses of these uncertain, small numbers often lead to significant differences. For example, we found 100% agreement for chromium due to its extremely low concentrations (median = 0.00001 µg/m^3^) estimated by NATA, while the 2005 NATA evaluation reported a 0% agreement when just using ratios [48]. This and other examples suggest ignorance of measurement uncertainty and, in particular, the LOQs, would lead to distinctly different results.

Previous studies have applied a number of model-to-monitor comparison metrics and methods, including biases and root mean square error [50,51,53,54], Kendall rank correlation [38], ratios [33,34,35], regressions [50], and even complex metrics [55]. There has been no commonly accepted criterion for the metric; for example, EPA uses a relative bias of within ±30% and median ratio of 0.5–2 for agreement. The goodness-of-fit of a regression line, indicated by R^2^, is often arbitrary. The median ratio of modeled-to-monitored concentrations is the most commonly used metric; however, it may become extremely small or large when concentrations are too small to be practically quantifiable. The strengths of our approach were the consideration of measurement uncertainty and statistical comparisons with the commonly used 95% confidence interval criterion.

### 4.3. Study Limitations

We have also acknowledged limitations in data sources and the comparison methodology. We used all the available annual averages without considering the number of measurements or seasonality, in order to increase the sample sizes. A representative annual average should be calculated from data measured in at least two seasons [52]. AQS did not report the LOQ for each measurement, and thus we adopted LOQs from EPA’s major contract laboratory [43]. The use of a single LOD for all the measurements of a compound might have caused misclassification of ∆Ms, considering varying LOQs over time or by laboratories. This limitation also calls for inclusion of LOQs in future air quality data reporting. The analysis unit was census tract, a small geographic unit often used in environmental disparity and epidemiology research. NATA already admitted that NATA estimates were unreliable at the census tract level and discouraged uses of census tract data [52]. The lack of local information refrained us from explaining regional differences in model performance. For example, the poor agreement of halogenated compounds in Region 2 might be due to data quality in emissions and meteorological conditions as well as photochemical reactions. The underlying factors contributing to these regional differences need further inquiry and investigation.

### 4.4. Implications for Environmental Health Disparity Research

The environment plays a critical role in determining people’s health [56]. Environmental health disparity is the difference in health risks that people have when they experience both uneven exposure to various environmental risk factors and social inequality [57]. It is often examined at census tract level because census tract is considered a geographic area roughly representative for a neighborhood where the sociodemographic characteristics are homogeneous among a stable size (1500 housing units and 4000 people on average) of the population [58]. With easily available census-tract level sociodemographic data from census and exposure data from NATA, many studies have examined environmental disparities in HAP exposures and risks, and always found a significant association between HAP exposure and sociodemographic status [21,24,59]. Our evaluation indicates two major uncertainties in NATA data. First, a large portion of NATA estimates are so low that they fall below the LOQs. Accordingly, large uncertainties exist in estimating cancer risks from exposure to carcinogenic HAPs when applying the linear non-threshold dose-response relationship. Second, NATA modeling was unable to predict extremely high concentrations due to lack of information on local, intermittent and sporadic emissions. These two uncertainties may result in false strong disparity patterns observed in many studies based on NATA data. The overall model performance warrants future disparity studies be conducted with actual HAP monitoring data, in particular, when examining disparities at the local level.

## 5. Conclusions

This study provides an independent model-to-monitor assessment for census-tract level HAP concentrations modeled in the latest NATA. Significant portions of modeled concentrations (5–100%) fell below the limits of quantitation (LOQs), and less than 30% of quantifiable concentrations showed statistical agreement. Out of the 27 compounds examined, 14 compounds showed agreement at over 50% of sites. Underestimation of NATA estimates was predominant in non-agreement cases. The agreement was inconsistent in terms of chemical group or region and was impacted by the comparison methods. These findings generally concurred with those from previous national, state, and local NATA evaluation studies. The substantial non-agreements of NATA predictions with monitoring data signal cautions for environmental epidemiology and justice studies that utilize NATA modeling data.

## Figures and Tables

**Table 1:** Descriptive statistics of annual average concentrations (µg/m^3^) in NATA-AQS and NATA-All datasets.

Hazardous Air Pollutants (HAPs)	NATA-AQS						NATA-All					
	N	Mean	SD	Min	Med	Max	N	Mean	SD	Min	Med	Max
**Aromatic compounds**												
Benzene	274	0.79	0.47	0.09	0.69	3.02	74,034	0.70	0.43	0	0.63	7.50
Toluene	257	2.62	3.01	0.09	2.08	30.37	74,034	2.61	3.46	0	1.84	39.67
Styrene	236	0.05	0.16	1.1 × 10^−3^	0.02	1.57	75,023	0.02	0.06	0	0.02	6.72
Ethylbenzene	252	0.28	0.21	0.02	0.25	1.66	75,025	0.23	0.19	0	0.20	3.09
Cumene	113	0.01	0.03	3.8 × 10^−4^	1.3 × 10^−3^	0.2	75,013	1.9 × 10^−3^	0.01	0	8.5 × 10^−4^	1.98
Naphthalene	52	0.05	0.04	4.2 × 10^−3^	0.04	0.22	74,034	0.04	0.03	0	0.03	0.94
1,4-Dichlorobenzene	153	0.01	0.04	2.6 × 10^−5^	6.4 × 10^−4^	0.27	74,034	0.02	0.05	0	5.1 × 10^−4^	1.13
**Halogenated compounds**												
Methyl chloride	203	1.10	0.07	1.09	1.09	2.06	74,591	1.07	0.13	0	1.09	2.06
Vinyl chloride	240	4.6 × 10^−3^	0.02	1.5 × 10^−5^	3.2 × 10^−4^	0.15	74,034	6.2 × 10^−4^	3.7 × 10^−3^	0	2.2 × 10^−4^	0.73
Bromomethane	200	0.04	0.09	0.03	0.03	1.34	74,438	0.04	0.05	0	0.03	2.00
Ethylene dichloride	230	3.4 × 10^−3^	0.01	3.2 × 10^−5^	5.3 × 10^−4^	0.08	74,034	7.5 × 10^−4^	3.7 × 10^−3^	0	3.8 × 10^−4^	0.31
Chloroform	252	0.01	0.03	1.7 × 10^−7^	1.2 × 10^−3^	0.23	74,034	3.8 × 10^−3^	0.01	0	8.6 × 10^−4^	1.57
Trichloroethylene	247	0.02	0.04	4.0 × 10^−4^	0.01	0.44	74,034	0.02	0.05	0	0.01	5.49
1,1,2-Trichloroethane	185	4.1 × 10^−4^	1.2 × 10^−4^	3.9 × 10^−4^	3.9 × 10^−4^	0	74,871	3.9 × 10^−4^	1.7 × 10^−4^	0	3.9 × 10^−4^	0.01
Carbon tetrachloride	247	0.55	7.1 × 10^−4^	0.55	0.55	0.55	74,917	0.54	0.08	0	0.55	0.64
Tetrachloroethylene	252	0.09	0.17	1.5 × 10^−3^	0.03	1.12	74,034	0.11	0.24	0	0.03	5.07
**Carbonyl compounds**												
Formaldehyde	128	1.66	0.52	0.55	1.61	2.94	74,034	1.59	0.55	0	1.57	5.56
Acetaldehyde	133	1.95	0.55	0.91	1.85	3.28	74,034	1.94	0.66	0	1.88	4.15
Methyl isobutyl ketone	93	0.1	0.15	4.8 × 10^−3^	0.07	1.27	74,968	0.07	0.08	0	0.05	2.12
**Other compounds**												
Chromium VI (TSP)	27	3.4 × 10^−5^	3.6 × 10^−5^	2.3 × 10^−6^	2.1 × 10^−5^	1.4 × 10^−4^	74,034	3.9 × 10^−5^	8.0 × 10^−5^	0	2.1 × 10^−5^	4.4 × 10^−3^
Acrylonitrile	81	2.6 × 10^−3^	0.01	4.9 × 10^−6^	3.5 × 10^−4^	0.09	74,034	7.9 × 10^−4^	0.01	0	3.0 × 10^−4^	1.24
1,3-Butadiene	258	0.08	0.06	3.9 × 10^−3^	0.06	0.44	74,034	0.06	0.05	0	0.05	0.79
Carbon disulfide	93	0.05	0.34	0.01	0.01	3.27	74,906	0.01	0.05	0	0.01	4.91
*n*-Hexane	159	0.91	0.6	0.12	0.83	2.87	75,020	0.76	0.66	0	0.60	16.05
Methyl *tert*-butyl ether	127	1.1 × 10^−3^	0.01	3.6 × 10^−9^	2.0 × 10^−5^	0.12	73,815	1.5 × 10^−3^	0.01	0	7.4 × 10^−6^	0.31
2,2,4-Trimethylpentane	105	0.36	0.19	0.1	0.34	0.92	75,009	0.37	0.23	0	0.33	3.59
Lead (TSP)	51	1.5 × 10^−3^	1.5 × 10^−3^	9.5 × 10^−5^	1.0 × 10^−3^	0.01	74,034	1.0 × 10^−3^	1.5 × 10^−3^	0	6.8 × 10^−4^	0.10

Notes: HAPs—hazardous air pollutants; NATA-All–all census tracts NATA covered in the U.S.; NATA-AQS–census tracts both NATA and AQS covered in the U.S.; N–the number of census tracts; SD—Standard deviation; Min, Med, and Max—Minimum, Median, and Maximum.

**Table 2:** National statistics of HAP concentrations measured by AQS and modeled by NATA.

HAPs	N	LOQ µg/m^3^	∆M < LOQ? Y/N	ΔM < LOQ % of Sites	AQS					NATA					Wilcoxon Rank Test *p*-value
					Mean µg/m^3^	SD µg/m^3^	Med µg/m^3^	Max µg/m^3^	<LOQ %	Mean µg/m^3^	SD µg/m^3^	Med µg/m^3^	Max µg/m^3^	<LOQ %	
**Aromatic compounds**															
Benzene	274	0.06	Y	12.0	0.95	0.78	0.73	5.57	0.7	0.79	0.47	0.69	3.02	0.0	0.01
Toluene	257	0.10	N	8.2	1.75	1.27	1.41	6.98	0.4	2.62	3.01	2.08	30.37	0.4	<0.001
Styrene	236	0.04	N	45.3	0.14	0.19	0.07	1.45	35.6	0.05	0.16	0.02	1.57	81.4	<0.001
Ethylbenzene	252	0.05	Y	18.3	0.32	0.80	0.21	12.57	9.1	0.28	0.21	0.25	1.66	10.7	0.60 *
Cumene	113	0.50	Y	94.7	0.11	0.40	0.03	3.79	93.8	0.01	0.03	1.3 × 10^−3^	0.20	100	<0.001
Naphthalene	52	7.8 × 10^−4^	N	3.9	0.07	0.11	0.04	0.56	0.0	0.05	0.04	0.04	0.22	0.0	0.89 *
1,4-Dichlorobenzene	153	0.07	Y	62.8	0.18	0.68	0.05	7.40	63.4	0.01	0.04	6.4 × 10^−4^	0.27	94.1	<0.001
**Halogenated compounds**															
Methyl chloride	203	0.04	N	12.3	1.23	0.25	1.23	2.5	0.5	1.10	0.07	1.09	2.06	0.0	<0.001
Vinyl chloride	240	0.01	Y	91.7	0.01	0.04	0.00	0.51	89.6	4.6 × 10^−3^	0.02	3.2 × 10^−4^	0.15	95.0	0.01
Bromomethane	200	0.02	Y	59.5	0.02	0.04	0.01	0.46	69.5	0.04	0.09	0.03	1.34	0.0	<0.001
Ethylene dichloride	230	0.02	Y	54.8	0.07	0.34	0.02	4.73	53.0	3.4 × 10^−3^	0.01	5.3 × 10^−4^	0.08	95.7	<0.001
Chloroform	252	0.04	N	26.2	0.13	0.39	0.09	6.05	25.0	0.01	0.03	1.2 × 10^−3^	0.23	93.7	<0.001
Trichloroethylene	247	0.03	Y	72.1	0.11	1.07	0.01	16.85	62.8	0.02	0.04	0.01	0.44	76.9	<0.001
1,1,2-Trichloroethane	185	0.05	Y	93.5	0.01	0.02	0.00	0.15	93.5	4.1 × 10^−4^	1.2 × 10^−4^	3.9 × 10^−4^	1.8 × 10^−3^	100	0.03
Carbon tetrachloride	247	0.04	N	25.1	0.54	0.19	0.59	1.68	3.6	0.55	7.1 × 10^−4^	0.55	0.55	0.0	<0.001
Tetrachloroethylene	252	0.06	N	39.7	0.13	0.14	0.09	1.10	29.0	0.09	0.17	0.03	1.12	67.1	<0.001
**Carbonyl compounds**															
Formaldehyde	128	0.03	N	4.7	1.29	0.71	1.16	6.77	0.0	1.66	0.52	1.61	2.94	0.0	<0.001
Acetaldehyde	133	0.03	N	5.3	1.56	0.63	1.44	3.73	0.0	1.95	0.55	1.85	3.28	0.0	<0.001
Methyl isobutyl ketone	93	0.08	Y	65.6	0.12	0.10	0.10	0.60	38.7	0.10	0.15	0.07	1.27	53.8	0.00
**Other compounds**															
ChromiumVI (TSP)	27	2.7 × 10^−3^	Y	100.0	1.3 × 10^−5^	1.3 × 10^−5^	1.2 × 10^−5^	4.7 × 10^−5^	100	3.4 × 10^−5^	3.6 × 10^−5^	2.1 × 10^−5^	1.4 × 10^−4^	100	0.00
Acrylonitrile	81	0.10	Y	79.0	0.11	0.24	0.01	1.06	79.0	2.6 × 10^−3^	0.01	3.5 × 10^−4^	0.09	100	<0.001
1,3-Butadiene	258	0.02	Y	29.5	0.08	0.14	0.05	1.74	34.1	0.08	0.06	0.06	0.44	12.0	<0.001
Carbon disulfide	93	0.02	N	20.4	0.92	3.44	0.07	29.44	19.4	0.05	0.34	0.01	3.27	95.7	<0.001
*n*-Hexane	159	0.31	Y	43.4	0.81	0.75	0.57	4.39	19.5	0.91	0.60	0.83	2.87	16.4	<0.001
Methyl *tert*-butyl ether	127	0.16	Y	98.4	0.01	0.07	0.00	0.72	98.4	1.1 × 10^−3^	0.01	2.0 × 10^−5^	0.12	100	0.08 *
2,2,4-Trimethylpentane	105	0.28	Y	79.1	0.50	0.53	0.34	3.1	39.0	0.36	0.19	0.34	0.92	38.1	0.16 *
Lead (TSP)	51	2.6 × 10^−4^	N	7.8	0.01	0.01	3.4 × 10^−3^	0.07	7.8	1.5 × 10^−3^	1.5 × 10^−3^	1.0 × 10^−3^	0.01	5.9	<0.001

Notes: HAPs—hazardous air pollutants; LOQ—limit of quantitation; SD—standard deviation; Med—median; Max—maximum. * *p*-value of >0.05 indicating no significant difference.

**Table 3:** Percentages of agreement, underestimation, and overestimation sites determined by NATA-AQS comparisons.

HAPs	N	ΔM < LOQ	Within 95% CL	Total Agreement	Less than 95% LCL (Under)	Greater than 95% UCL (Over)
**Aromatic compounds**						
Benzene	274	12.0	9.9	21.9	43.8	34.3
Toluene	257	8.2	20.6	28.8	18.7	52.5 *
Styrene	236	45.3	5.9	51.3 *	45.3	3.4
Ethylbenzene	252	18.3	9.5	27.8	36.5	35.7
Cumene	113	94.7	0.0	94.7 *	5.3	0.0
Naphthalene	52	3.8	26.9	30.8	30.8	38.5
1,4-Dichlorobenzene	153	62.7	3.3	66.0 *	30.7	3.3
**Halogenated compounds**						
Methyl chloride	203	12.3	10.3	22.7	63.1 *	14.3
Vinyl chloride	240	91.7	2.9	94.6 *	5.0	0.4
Bromomethane	200	59.5	3.5	63.0 *	2.5	34.5
Ethylene dichloride	230	54.8	3.5	58.3 *	41.3	0.4
Chloroform	252	26.2	5.2	31.3	68.7 *	0.0
Trichloroethylene	247	72.1	4.9	76.9 *	15.0	8.1
1,1,2-Trichloroethane	185	93.5	1.6	95.1 *	4.9	0.0
Carbon tetrachloride	247	25.1	2.8	27.9	49.8 *	22.3
Tetrachloroethylene	252	39.7	13.9	53.6 *	29.8	16.7
**Carbonyl compounds**						
Formaldehyde	128	4.7	21.1	25.8	7.0	67.2 *
Acetaldehyde	133	5.3	21.8	27.1	10.5	62.4 *
Methyl isobutyl ketone	93	65.6	3.2	68.8	23.7	7.5
**Other compounds**						
Chromium VI (TSP)	27	100.0	0.0	100.0 *	0.0	0.0
Acrylonitrile	81	79.0	1.2	80.2 *	19.8	0.0
1,3-Butadiene	258	29.5	10.5	39.9	18.2	41.9
Carbon disulfide	93	20.4	17.2	37.6	60.2 *	2.2
*n*-Hexane	159	43.4	5.0	48.4	13.2	38.4
Methyl *tert*-butyl ether	127	98.4	0.0	98.4 *	1.6	0.0
2,2,4-Trimethylpentane	105	79.0	0.0	79.0 *	17.1	3.8
Lead (TSP)	51	7.8	5.9	13.7	74.5 *	11.8

Notes: LOQ—limit of quantitation; CI—confidence interval; LCL—lower confidence limit; UCL—upper confidence limit; Under—underestimation; Over—overestimation. * Percentages of agreement, underestimation, and overestimation sites are ≥50%.

**Table 4:** Percentages of agreement, underestimation, and overestimation sites determined by NATA-AQS comparisons using the fractional bias method.

HAPs	N	Fractional Bias				Total Agreement
		ΔM < LOQ	(−2, −0.67)	(−0.67, 0.67)	(0.67, 2)	
		%				
**Aromatic compounds**						
Benzene	274	12.0	20.1	55.8	12.0	67.9 *
Toluene	257	8.2	11.7	48.2	31.9	56.4 *
Styrene	236	45.3	49.2	2.5	3.0	47.9
Ethylbenzene	252	18.3	19.4	40.1	22.2	58.3 *
Cumene	113	94.7	5.3	0	0	94.7 *
Naphthalene	52	3.8	21.2	55.8	19.2	59.6 *
1,4-Dichlorobenzene	153	62.7	34.0	1.3	2.0	64.1 *
**Halogenated compounds**						
Methyl chloride	203	12.3	0.5	85.7	1.5	98.0 *
Vinyl chloride	240	91.7	6.3	2.1	0	93.8 *
Bromomethane	200	59.5	4.0	2.0	34.5	61.5 *
Ethylene dichloride	230	54.8	44.8	0.4	0	55.2 *
Chloroform	252	26.2	71.8	2.0	0	28.2
Trichloroethylene	247	72.1	18.6	2.0	7.3	74.1 *
1,1,2-Trichloroethane	185	93.5	6.5	0	0	93.5 *
Carbon tetrachloride	247	25.1	0.4	63.6	10.9	88.7 *
Tetrachloroethylene	252	39.7	42.5	4.8	13.1	44.4
**Carbonyl compounds**						
Formaldehyde	128	4.7	1.6	78.1	15.6	82.8 *
Acetaldehyde	133	5.3	0	81.2	13.5	86.5 *
Methyl isobutyl ketone	93	65.6	23.7	4.3	6.5	69.9 *
**Other compounds**						
Chromium VI (TSP)	27	100.0	0	0	0	100 *
Acrylonitrile	81	79.0	21.0	0	0	79.0 *
1,3-Butadiene	258	29.5	18.6	16.7	35.3	46.1
Carbon disulfide	93	20.4	75.3	2.2	2.2	22.6
*n*-Hexane	159	43.4	8.8	20.8	27.0	64.2 *
Methyl *tert*-butyl ether	127	98.4	1.6	0	0	98.4 *
2,2,4-Trimethylpentane	105	79.0	13.3	5.7	1.9	84.8 *
Lead (TSP)	51	7.8	68.6	13.7	9.8	21.6

Notes: * The percentage of the total agreement is ≥50%.
